# Dynamic strain scanning optimization: an efficient strain design strategy for balanced yield, titer, and productivity. DySScO strategy for strain design

**DOI:** 10.1186/1472-6750-13-8

**Published:** 2013-02-06

**Authors:** Kai Zhuang, Laurence Yang, William R Cluett, Radhakrishnan Mahadevan

**Affiliations:** 1 Department of Chemical Engineering and Applied Chemistry, University of Toronto, 200 College Street, Toronto, ON, M5S 3E5, Canada; 2 Institute of Biomaterials and Biomedical Engineering, University of Toronto, 164 College Street, Toronto, ON, M5S 3G9, Canada

**Keywords:** Metabolic modeling, Process modeling, Strain design, Dynamic strain design

## Abstract

**Background:**

In recent years, constraint-based metabolic models have emerged as an important tool for metabolic engineering; a number of computational algorithms have been developed for identifying metabolic engineering strategies where the production of the desired chemical is coupled with the growth of the organism. A caveat of the existing algorithms is that they do not take the bioprocess into consideration; as a result, while the product yield can be optimized using these algorithms, the product titer and productivity cannot be optimized. In order to address this issue, we developed the Dynamic Strain Scanning Optimization (DySScO) strategy, which integrates the Dynamic Flux Balance Analysis (dFBA) method with existing strain algorithms.

**Results:**

In order to demonstrate the effective of the DySScO strategy, we applied this strategy to the design of *Escherichia coli* strains targeted for succinate and 1,4-butanediol production respectively. We evaluated consequences of the tradeoff between growth yield and product yield with respect to titer and productivity, and showed that the DySScO strategy is capable of producing strains that balance the product yield, titer, and productivity. In addition, we evaluated the economic viability of the designed strain, and showed that the economic performance of a strain can be strongly affected by the price difference between the product and the feedstock.

**Conclusion:**

Our study demonstrated that the DySScO strategy is a useful computational tool for designing microbial strains with balanced yield, titer, and productivity, and has potential applications in evaluating the economic performance of the design strains.

## Background

Many microorganisms contain native pathways capable of producing useful chemical compounds [1,2], and synthetic pathways can be genetically inserted into model organisms such as *E. coli*[3] and *S. cerevisiae*[4]. Unfortunately, from the microorganisms’ perspective, most of these chemicals are metabolic byproducts, and their production is often minimal in wild-type organisms [2]. To create viable microbial cell factories, the production of these chemicals must be enhanced. Traditionally, biochemical production is enhanced through the mechanism of serial mutagenesis and phenotypic selection [5]. The advent of metabolic engineering introduced an new strain development approach in which genes are either amplified or deleted based on systematic consideration of the metabolic network, often with the aid of constraint-based metabolic models [2,5]. A caveat of metabolic engineering is that the outcome of strain improvement is limited by its scope: because metabolic engineering is focused on the cellular metabolism, it often neglects the overall bioprocess [5].

One approach to metabolic engineering is to couple the production of the desired chemical to the growth of the cell, thus ensuring the production of the chemical [6,7]. A number of constraint-based computational algorithms have been developed for identifying growth-coupled strain designs that enhance the product yield. Whereas several of these algorithms, such as OptKnock [8] and GDLS [9], focuses on identifying gene knockouts only, other algorithms, such as OptReg [10] and OptReg’LS [11] are capable of identifying reaction activation [12] and inhibition [13] as well. More recent advances include OptForce, which maximizes product yield by knockout, inhibition, and activation targets, relative to a wild-type flux distribution [14], and EMILiO, which rapidly identifies the optimal set of modified reactions and their optimal fluxes using a successive linear programming procedure [11]. The application of such algorithms have led to the development of industrial microbial cell factories [3].

Unfortunately, the limitations of metabolic engineering without process engineering are evident in these algorithms: while these algorithms can optimize the product yield of the strain, they cannot optimize the productivity and titer of the strain because they are process-level concepts and cannot be predicted using standard metabolic models. This is problematic because the economic viability of a bioprocess is commonly evaluated by its product yield, titer, and productivity [15,16]. Similarly, titer and productivity may also influence other high-level design objectives such as the ecological benefits of biochemical production. Most existing algorithms seek enhancements in product yield and growth rate, with the hopeful assumption that by improving these two attributes, titer and productivity will also improve. However, since the feedstock in a bioprocess can be converted into either biomass or desired product, the growth yield and the product yield cannot be simultaneously maximized. At the same substrate uptake rate, a higher growth yield will lead to a higher growth rate at the expense of the product yield. Constrained by this tradeoff, previous strain-design efforts often prioritize product yield optimization by restricting the growth rate to an arbitrarily low level [8,11,17-19]. However, a strain with a reduced growth rate would yield lower biomass concentration in bioreactors, which may reduce the volumetric productivity despite the increase in product yield. Recognizing this bias, Feist *et al.* (2010) have included substrate specific productivity (SSP) as an additional design criterion [2]. However, whereas SSP increases linearly with growth rate, productivity increases exponentially with growth rate. As such, SSP cannot replace productivity as a design criterion.

Fortunately, it is possible to predict titer and productivity using dynamic flux balance analysis (dFBA) – which incorporates both the process dynamics and the metabolic network [20-25]. By integrating the existing strain-design algorithms with dFBA, we have developed a novel strain-design strategy called Dynamic Strain Scanning Optimization (DySScO). By using product yield, titer, and volumetric productivity as explicit design criteria, DySScO can be used, potentially, to optimize high-level design objectives such as economic viability and ecological benefits. In this article, we present the rationale behind the DySScO strategy as well as the applications of this approach in designing *E. coli* strains for succinate and 1,4-butanediol production.

## Method

### An algorithmic description of the DySScO strategy

**Figure 1:**
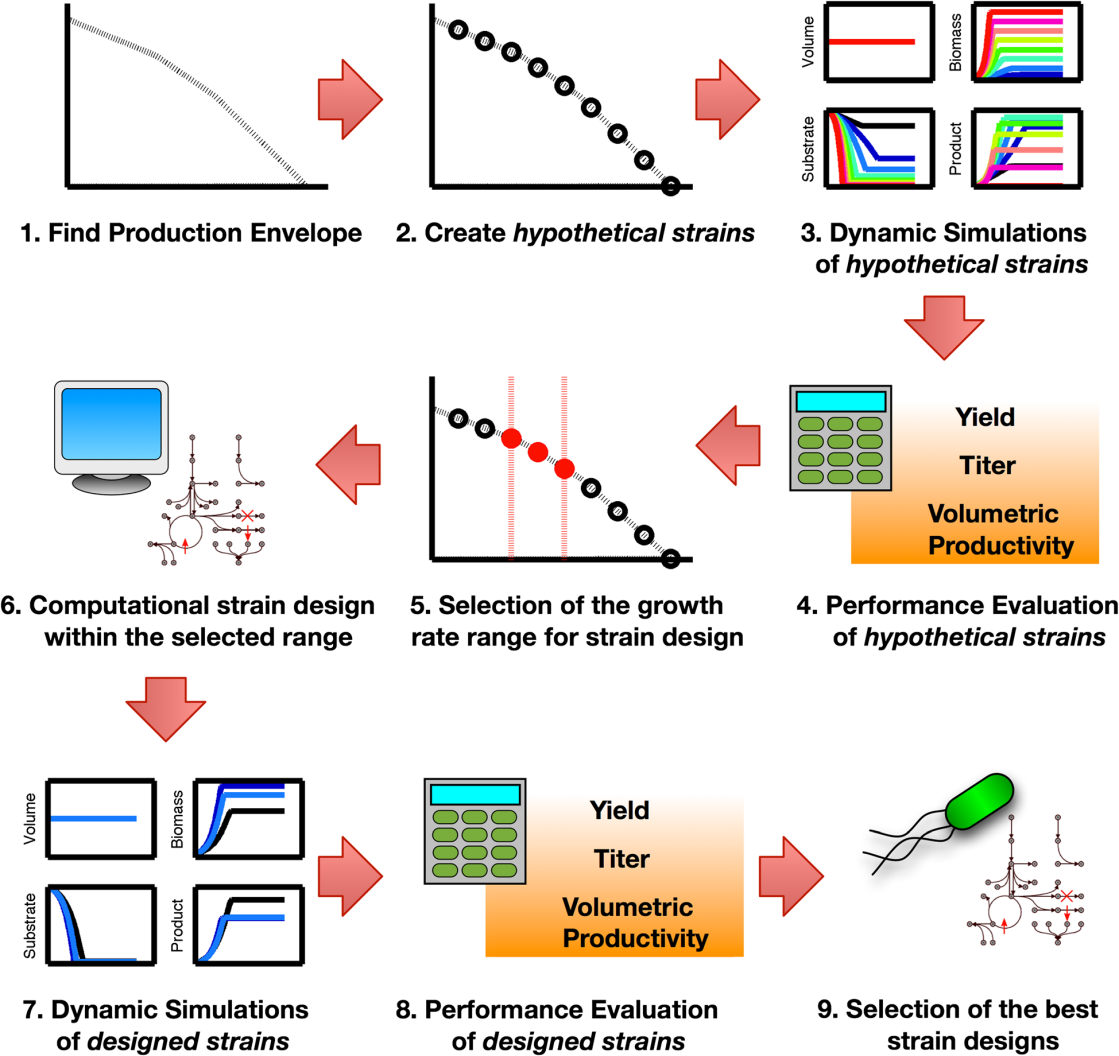
**A schematic diagram of the DySScO strategy.** The DySScO strategy is divided into three phases, which is subdivided into 9 steps. The “scanning” phase includes steps 1–5. The “design” phase include step 6. The “selection” phase includes steps 7–9.

The DySScO strategy consists of three major phases: scanning, design, annd selection. Here, these phases are broken down further and presented as 9 algorithmic steps (Figure 1). Many of the individual steps can be accomplished using a variety of algorithms and tools. For example, Step 6 can be performed using several existing strain design algorithm, such as OptKnock, GDLS, OptReg, OptReg’LS, and EMILiO.

1. Find the production envelope for the desired product upon assuming a fixed specific substrate uptake rate. The production envelope is the Pareto frontier in the product flux (mmol/gdw/hr) *vs.* biomass flux (gdw/gdw/hr) plane.

2. Create N hypothetical flux distributions along the upper bound of the product envelope by fixing their yield. In this work, the production envelope was generated using the COBRA Toolbox for Matlab, and N = 10 is used. The first strain had the highest yield, but did not grow; this strain is clearly not a viable solution and was not simulated.

3. Perform dynamic simulations of the behaviors of the hypothetical flux distributions inside bioreactors (eg. batch, fedbatch) using dFBA. In this work, the DyMMM framework is used for dFBA simulations, and ODE45 is used for simulating the dFBA equations.

4. The performance of the hypothetical flux distributions are evaluated using product yield (Y), titer (T), and volumetric productivity (P) calculated from the dynamic simulations. The performance of the strains can be ranked using any metric of form:

(1) $$Z=f\left(Y,T,P\right)$$

In our work, we used a simple metric called the consolidated strain performance (CSP), which is calculated as the weighted normalized sum of Y, T, and P:

(2) $$\begin{array}{c} \mathrm{CSP}=W_{1}*{Y/Y}_{\max}+W_{2}*{T/T}_{\max}\\ \;+W_{3}*{P/P}_{\max} \end{array}$$

5. Based on the performance of the hypothetical flux distributions, the growth rate range for the static strain design process is selected.

6. Existing strain design algorithms are used to find a number of high product yield designed strains within the optimal growth rate range. In this work, the GDLS algorithm is used. Experimental expertise and knowledge can be used here to eliminate predicted strain designs that are impossible to implement.

7. The dynamic behaviors of the designed strains inside bioreactor (eg. batch, fedbatch) are simulated using dFBA.

8. The performances of the designed strains are evaluated similar to step 4.

9. The best strain design is selected from the set of designed strains based on their CSP.

### Application of DySScO: succinate and BDO production in E. Coli

The general DySScO steps were followed in order to design both the succinate-producing strain and the BDO-producing strain. The *E. coli* iAF1260 metabolic model is used as the base model; the BDO biosynthesis pathway developed by Genomatica was added to this model in the BDO case [3].

### dFBA simulations using DyMMM framework

The dynamic multi-species metabolic modeling (DyMMM) framework [26] is a previously described extension to dFBA [20-23,27] designed to model the dynamic interactions between multiple microbial species. However, if only one species is present, then the DyMMM framework mathematically reduces to its predecessor dFBA. We have adapted the DyMMM framework for our dFBA simulations of the hypothetical flux distributions and designed strains because it is much more flexible than the native dFBA function provided by the COBRA Toolbox. Specifically, we have modified the DyMMM to track the volume of the reactor content (by adding Equation 3a), thus enabling the modeling of fedbatch reactors. In brief, the modified DyMMM framework can be mathematically described as:

(3a) $$\frac{\mathrm{dV}}{\mathrm{dt}}=F_{\mathrm{in}}-F_{\mathrm{out}}$$

(3b) $$\frac{dX_{i}}{\mathrm{dt}}=\mu_{i}X_{i}-\frac{F_{\mathrm{in}}X_{i}}{V}$$

(3c) $$\frac{dS^{j}}{\mathrm{dt}}=\sum_{i}v_{i}^{j}X_{i}+\frac{F_{\mathrm{in}}\left(S_{\mathrm{feed}}^{j}-S^{j}\right)}{V}$$

(3d) $$\begin{array}{l} \max\;c^{T}\bar{v}\\ \begin{array}{cc} \mathrm{st}. & \;A\bar{v}=0\\ \; & {\bar{v}}_{\min}<\bar{v}<{\bar{v}}_{\max} \end{array} \end{array}$$

Here, V is the volume (L) of the reactor, X_i_ is the biomass (g/L) of the i^th^ microbial species, S^j^ is the concentration (mM) of the j^th^ metabolite, F_in_ is the rate of flow (L/hr) into the reactor, F_out_ is the rate of flow (L/hr) out of the reactor, and S^j^_feed_ is the concentration of the j^th^ metabolite in the feed stream. μ_i_ is the growth rate of the ith microbial species, and v_i_^j^ is j^th^ the metabolic flux of the i^th^ microbial species; μ_i_ and v_i_^j^ are calculated from FBA (Equation 3d).

### Simulation parameters and reactor setup

We have assumed that both batch and fedbatch fermentations are carried out in fully anaerobic condition. We have assumed that all *E. coli* strains modeled have a maximum glucose uptake of 20 mmol/gdw/hr [28,29] and a Michaelis half-saturation constant (K_m_) of 1 mM under anaerobic conditions.

For batch simulations, the reactor is assumed to be well mixed at all times. The initial biomass is set to 0.01 g/L, the initial glucose concentration is set to 20 mM, and the initial liquid volume is set to 1 L. The batch time is set to 50 hours.

For fedbatch simulations, the reactor is assumed to be well mixed at all times. The initial biomass is set to 0.01 g/L, the initial glucose concentration is set to 20 mM, and the initial liquid volume is set to 1 L. The fedbatch time is set to 150 hours. The reactor liquid volume is limited to 10 L. An idealized exponential feeding strategy is used where glucose is continuously added to the bioreactor in order to maintain the glucose concentration at 20 mM until the reactor liquid volume reaches 10 L [30]. The glucose feed concentration is set to 1000 mM. The rate of glucose addition is governed by the following equation:

(4) $$Q_{\mathrm{in}}^{\mathrm{glc}}=-v^{\mathrm{glc}}\mathrm{XV}/\left(S_{\mathrm{feed}}^{\mathrm{glc}}-S^{\mathrm{glc}}\right)$$

Here, Q^glc^_in_ is the glucose feed rate (mM/hr), v^glc^_s_ is biomass specific glucose consumption rate (mmol/gdw/hr), X is the biomass concentration (g/L), V is liquid volume (L), S^glc^_feed_ (mM) is the glucose concentration in the feed stream, and Sglc (mM) is the glucose concentration in the reactor.

The simulation setups are the same for both succinate and BDO cases.

### Modeling succinate inhibition

The product inhibition of *E. coli* metabolism by succinate is modeled using a formulation that is similar to the extended Monod formulation used by Lin *et al.* to modeled the succinate inhibition of *Actinobacillus succinogenes*[31].

(5) $$v^{\mathrm{glc}}\leq v_{\max}^{\mathrm{glc}}\;{\left(1-\frac{S^{\mathrm{succ}}}{S^{\mathrm{succ},*}}\right)}^{k}\left(\frac{S^{\mathrm{glc}}}{S^{\mathrm{glc}}+K_{m}}\right)$$

Here, S^succ,*^ is the critical succinate concentration at which *E. coli* stops growing. Based on the work of Li *et al.* (2010) on four *E. coli* strains [32], we estimated S^succ,*^ to be 80g/L or 678mM of succinate. k is a fitting parameter that determines that shape of the inhibition curve, which we assumed to be at 1.5.

To check the strain’s sensitivity to a stronger inhibition effect, we also simulated the scenario where S^succ,*^ is halved to 40g/L or 339mM.

### Calculating yield, titer, and productivity

The yield (Y), titer (T), and productivity (P) is calculated as follows:

(6) $$\begin{array}{c} Y=\left(S_{f}^{\mathrm{target}}V_{f}\right)/\left(S_{o}^{\mathrm{feedstock}}V_{o}{-S}_{f}^{\mathrm{feedstock}}V_{f}\right)\\ \;{+S}_{\mathrm{feed}}^{\mathrm{feedstock}}\left(V_{f}{-V}_{o}\right) \end{array}$$

(7) $${T=S}_{f}^{\mathrm{target}}$$

(8) $${P=S}_{f}^{\mathrm{target}}{/t}_{f}$$

Here, subscript 0 indicate the beginning time, subscript f indicate the finish time. S^feedstock^_feed_ is the concentration of the feedstock in the feed stream; this value is reduced to zero in batch reactors.

### The analysis of the economic performance of the strains

The economic performance of the hypothetical flux distributions and designed strains in batch reactors were analyzed using two metrics: the hourly profit (Z_h_), which is the profit made per hour, and the batch profit (Z_b_), which is the profit made per batch. These metrics are calculated using the equations below:

(9) $$Z_{h}{=p}^{\mathrm{product}}\times {P-p}^{\mathrm{feedstock}}\times P/Y$$

(10) $$Z_{b}=p^{\mathrm{product}}\times {\mathrm{T-p}}^{\mathrm{feedstock}}\times S_{0}^{\mathrm{feedstock}}$$

Here, p^product^ is the price of the product, p^feedstock^ is the price of the feedstock, P is the volumetric productivity, Y is the product yield, T is the titer, and S_0_^feedstock^ is the concentration of the feedstock at the start of the batch. These calculations do not take the downstream costs (*eg.* seperation cost) and facility costs into consideration. The price of glucose (p^feedstock^) is assumed to be $0.13/mol, which is acquired from US Department of Agriculture’s Economic Research Service’s website (http://ers.usda.gov/data-products/sugar-and-sweeteners-yearbook-tables.aspx). The Z_b_ and Z_h_ of both succinate and BDO strains are evaluated at three price points: when the price of the product is 2X, 3X, and 8X of the price of glucose. The Z_h_ formulation is based on the economic formulation of chemical process found in the literature [33].

### Strain design using GDLS

The GDLS (Genetic Design through Local Search) algorithm [9]was used to identify knockout strategies for succinate and BDO over-production in *E. coli*, using the iAF1260 genome-scale model. For each iteration of GDLS, we used a neighborhood size of 2, and a single search path. In addition, we implemented constraints to prevent the local search from cycling back to the previous solution. Each MILP problem (i.e., local search iteration) was given a timeout threshold of 1800 seconds. If the MILP problem did reach the timeout threshold, then GDLS was continued only if a feasible, but not necessarily optimal, solution was identified. In this work, every local search MILP indeed found a feasible solution even if the timeout threshold was met. The MILPs were solved using CPLEX 12.1 and the CPLEXINT interface, with up to 8 parallel threads using 2.4 GHz AMD Opteron processors.

## Results and discussions

### Dynamic strain scanning optimization (DySScO) strategy

The Dynamic Strain Scanning Optimization (DySScO) Strategy is a novel strain design strategy capable of producing strains with optimized product yield, titer, and volumetric productivity. DySScO consists of three major phases: scanning, design, and selection. First, during the “scanning” phase, by simulating the dynamic behaviors of a large number of hypothetical flux distributions along the upper boundary of the production envelope using dFBA, an optimal range of growth rates can be determined based on the desired design criteria. Then, during the “design” phase, existing strain-design algorithms (e.g. OptKnock, GDLS, EMILiO) can be used to find the potential strains with high product yield within this growth rate range. Lastly, during the “selection” phase, dFBA simulations of these potential strains are performed and the best strain-design is selected based on the desired design criteria.

In order to rank the performance of the strains, any design criterion that is a function of the yield, titer, and volumetric productivity can be used. In our work, we introduced a metric called consolidated strain performance (CSP), which is a weighted sum of the normalized product yield, titer, and volumetric productivity (Equation 2). Since the present work is a proof of principle study of the DySScO strategy, we assigned equal weights of 1 to the three design criteria. This weighting system is good for generating strain designs with balanced yield, titer, and volumetric productivity. In real world applications, the weights should be determined on a case-by-case basis based on higher level design objectives such as the relative economic values or ecological benefits.

In order to demonstrate the necessity and the effectiveness of the DySScO strategy, we applied the strategy to two strain design problems: the designing of a succinate-producing *E. coli* strain and an 1,4-butanediol (BDO) producing *E. coli* strains.

### Designing succinate-producing strains

Since Succinate is a commodity chemical that is naturally produced by *E. coli*, many previous metabolic engineering efforts have sought to design succinate-producing *E. coli* strains. We applied DySScO to the designing of succinate-producing *E. coli* strain in order to demonstrate the effectiveness of this strategy, and distinguish it from previous strain-design strategies.

**Figure 2:**
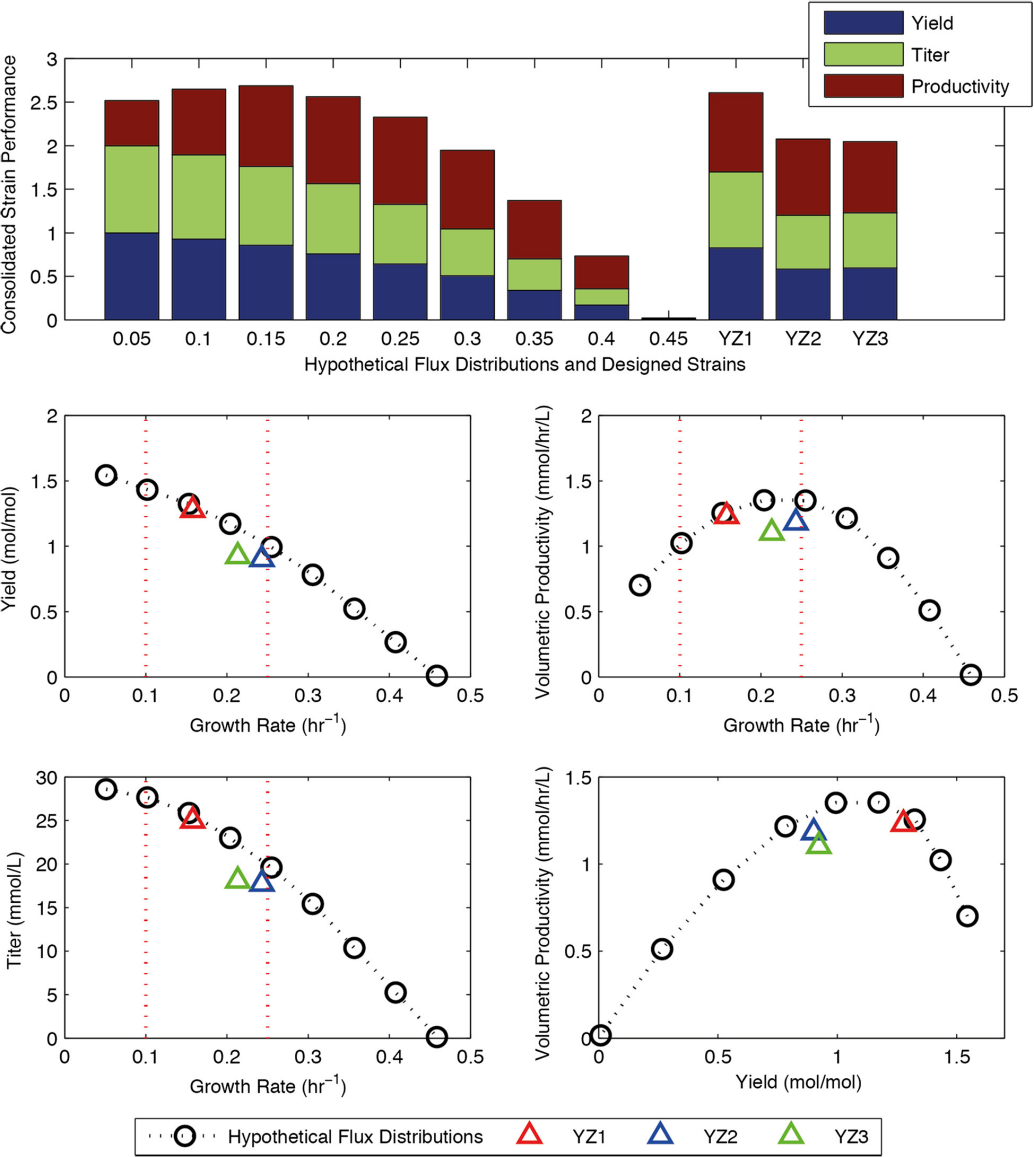
**The performance of the succinate-producing strains in batch. (A-D)** The consolidated strain performance **(A)**, succinate yield **(B)**, volumetric productivity **(C)**, and titer **(D)** of both the hypothetical flux distributions and the designed strains in batch simulations. The red dotted line indicates the range of growth rates selected for the static strain design process. **(E)** The tradeoff between the succinate yield and the volumetric productivity. The pareto frontier is outlined by the dotted line connecting the hypothetical flux distributions.

During the “scanning” phase, ten hypothetical flux distributions were created *in silico* using the iAF1260 model of *E. coli.* By constraining the succinate flux, these strains were constrained to the upper boundary of the succinate production envelope (Figure 2B). dFBA was used to simulate the dynamic behaviors of these strains in batch reactors, and the maximum-achievable yield, titer, and productivity of these strains were calculated from the predicted process dynamics (Figure 2A-D). Amongst the ten hypothetical flux distributions, the flux distribution with the growth rate of 0.15 hr^−1^ has the best overall performance (Figure 2A). We rejected the strain growing at the rate of 0.05 hr^−1^ because it strongly favored yield and neglected productivity. Based on these results, we selected a growth range of 0.1-0.25 hr^−1^ for the strain design process (Figure 2A).

During the “design phase”, the GDLS strain design algorithm [9] was used to generate a set of knockout strains that coupled succinate production to biomass synthesis (see Methods). Three strains were selected from the list of strains produced, hereby designated as “YZ1”, “YZ2”, and “YZ3”. YZ1 has a growth rate of 0.16 hr^−1^, and is very close to the production envelope, while strains YZ2 and YZ3 have growth rates of 0.24 hr^−1^ and 0.21 hr^−1^, respectively, and are slightly farther from the production envelope (Table 1, Figure 2B).

**Table 1:** Knockout strategies for succinate overproduction identified in this work

**Succinate Strains**	**YZ1**	**YZ2**	**YZ3**
Growth rate (hr^-1^)	0.16	0.24	0.21
Product yield	1.27	0.89	0.92
(mol/mol glc)	ALCD2x	F6PA	ACALD
Knockouts	GLUDy	G6PDH2r	F6PA
	LDH_D	ME2	G6PDH2r
	PPKr	MTHFD	GLUDy
	TKT2	PFL	ME2
		PYK	PFL
			PYK

The knockouts are listed by their reaction names in the iAF1260 genome-scale model of *E. coli* metabolism.

The specific knockout strategies used in the strains YZ1, YZ2, and YZ3 are listed in Table 1. Many of the knockouts identified in this work (Table 1) overlapped with those found in the literature. For example, the knockout of pyruvate formate lyase (PFL), alcohol dehydrogenase (ALCD2x), and D-lactate dehydrogenase (LDH_D) is consistent with the common experimental strategy [34] where the pathways for fermentation products (eg. formate, ethanol, lactate) are knocked out. The knockout of the NADP-dependent malic enzyme (ME2) or glucose-6-phosphate dehydrogenase (G6PDH2r) is consistent with previously identified *in silico* strategies[2]. This overlap is not surprising since DySScO utilizes existing strain-design algorithms to identify the list of potential strains; what distinguishes DySScO is that the “best strain” is selected by ranking them based on an equally weighted consolidated strain performance score, thus achieving a balance between the three design criteria.

**Figure 3:**
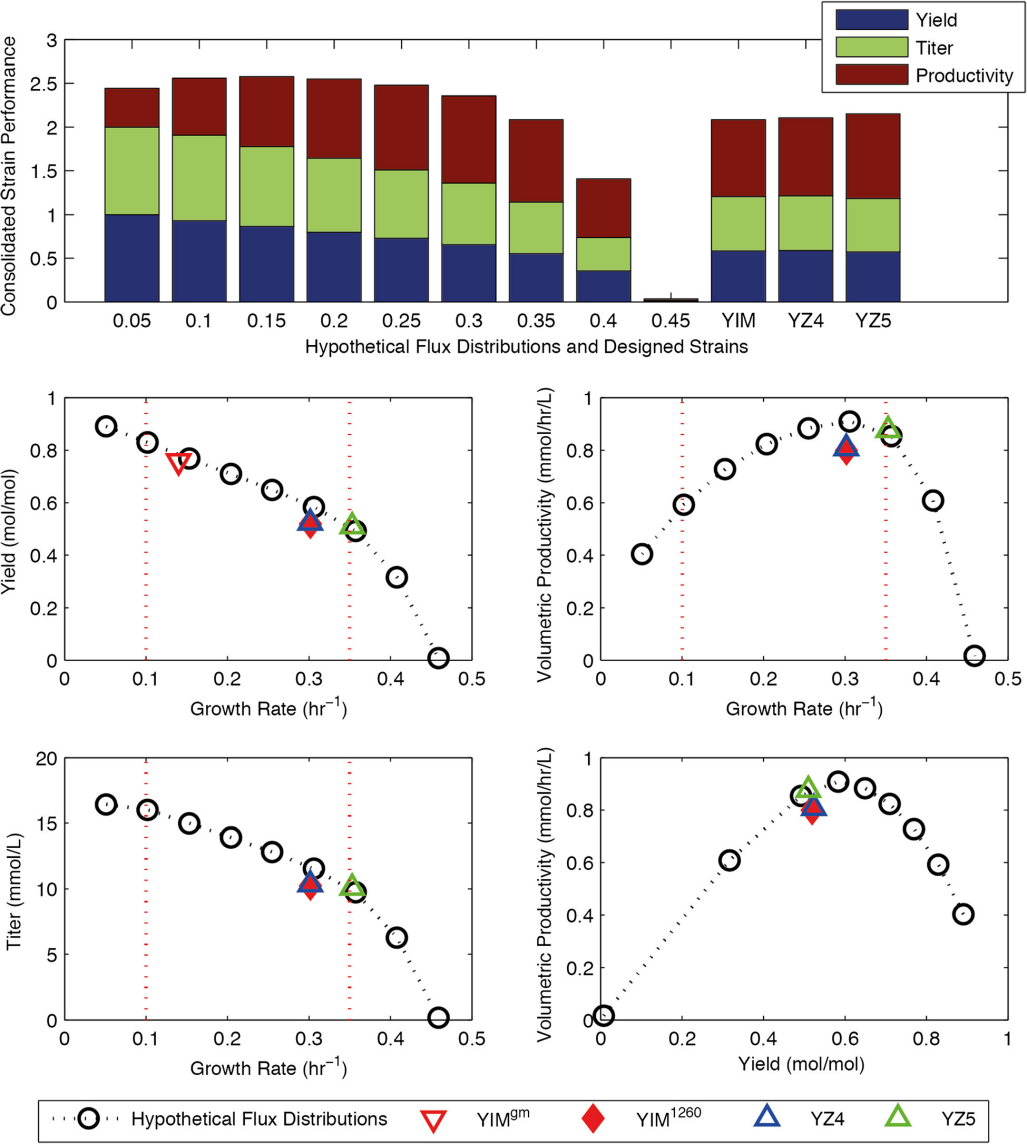
**The economic performance of the succinate-producing strains.** The consolidated strain performance **(A, B, C)**, hourly profit **(D, E, F)**, and profit per batch **(G, H, I)** of the succinate-producing strains at three different succinate price points. The horizontal dotted line **(D-I)** indicates the zero profit line.

During the “selection” phase, dFBA was used to predict the maximum-achievable yield, titer, and productivity of the strains YZ1, YZ2, and YZ3. The performances of these strains are compared with those of the hypothetical flux distributions (Figure 2A, Figure 3A-C). Both the overall performance (Figure 2A, Figure 3A-C) and the dynamics of YZ1 (not shown) are similar to that of the hypothetical flux distributions with the growth rate of 0.15 hr^−1^. It significantly outperforms YZ2, YZ3 (Figure 2A, Figure 3A-C) as it has a significantly higher volumetric productivity than the 0.1 hr^−1^ hypothetical flux distribution, while having only a slightly lower product yield. As such, we selected YZ1 to be the best strain using the consolidated performance metric.

### Designing 1,4-butanediol-producing strains

Previously, Yim *et al.* from Genomatica Inc. have reported a synthetic pathway in *E. coli* that allows for the fermentation of 1,4-butanediol (BDO), a non-native metabolite [3]. The OptKnock algorithm was used to recommend a number of growth-coupled BDO-producing strain designs, and a strain design predicted to have a high product yield (0.76 mol/mol) and a low growth rate (0.14 hr − 1) was chosen and implemented [3]. In the present work, we added this BDO-producing pathway to the iAF1260 model of *E. coli*, and applied DySScO in hope to find a better strain that is optimized for the consolidated strain performance.

**Figure 4:**
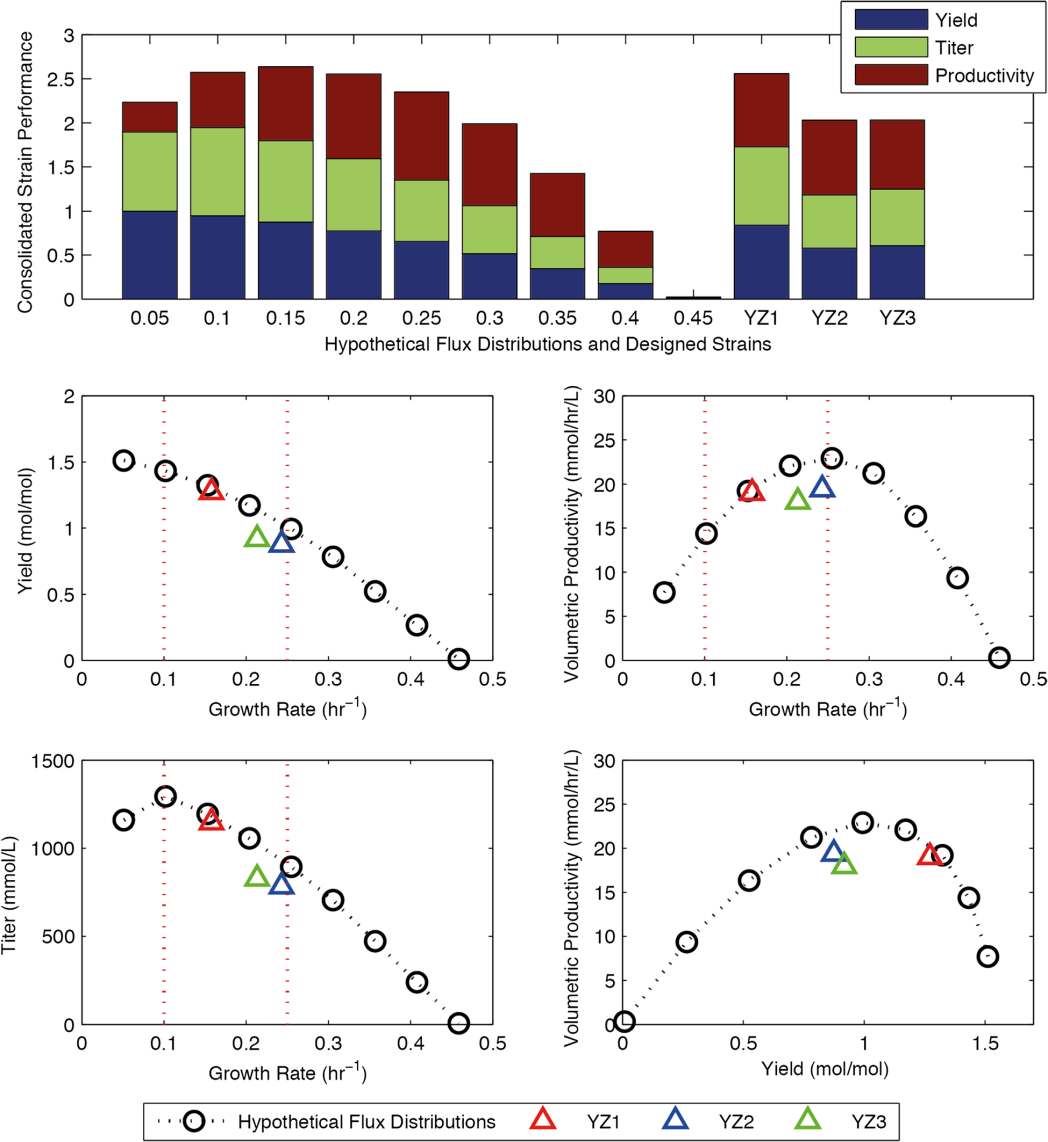
**The performance of the BDO-producing strains in batch. (A-D)** The consolidated strain performance **(A)**, BDO yield **(B)**, volumetric productivity **(C)**, and titer **(D)** of the hypothetical flux distributions, the designed strains, and the strain designed by Yim *et al.* (2011) in batch simulations. The red dotted line indicates the range of growth rates selected for the static strain design process. **(E)** The tradeoff between the BDO yield and the volumetric productivity. The pareto frontier is outlined by the dotted line connecting the hypothetical flux distributions.

**Figure 5:**
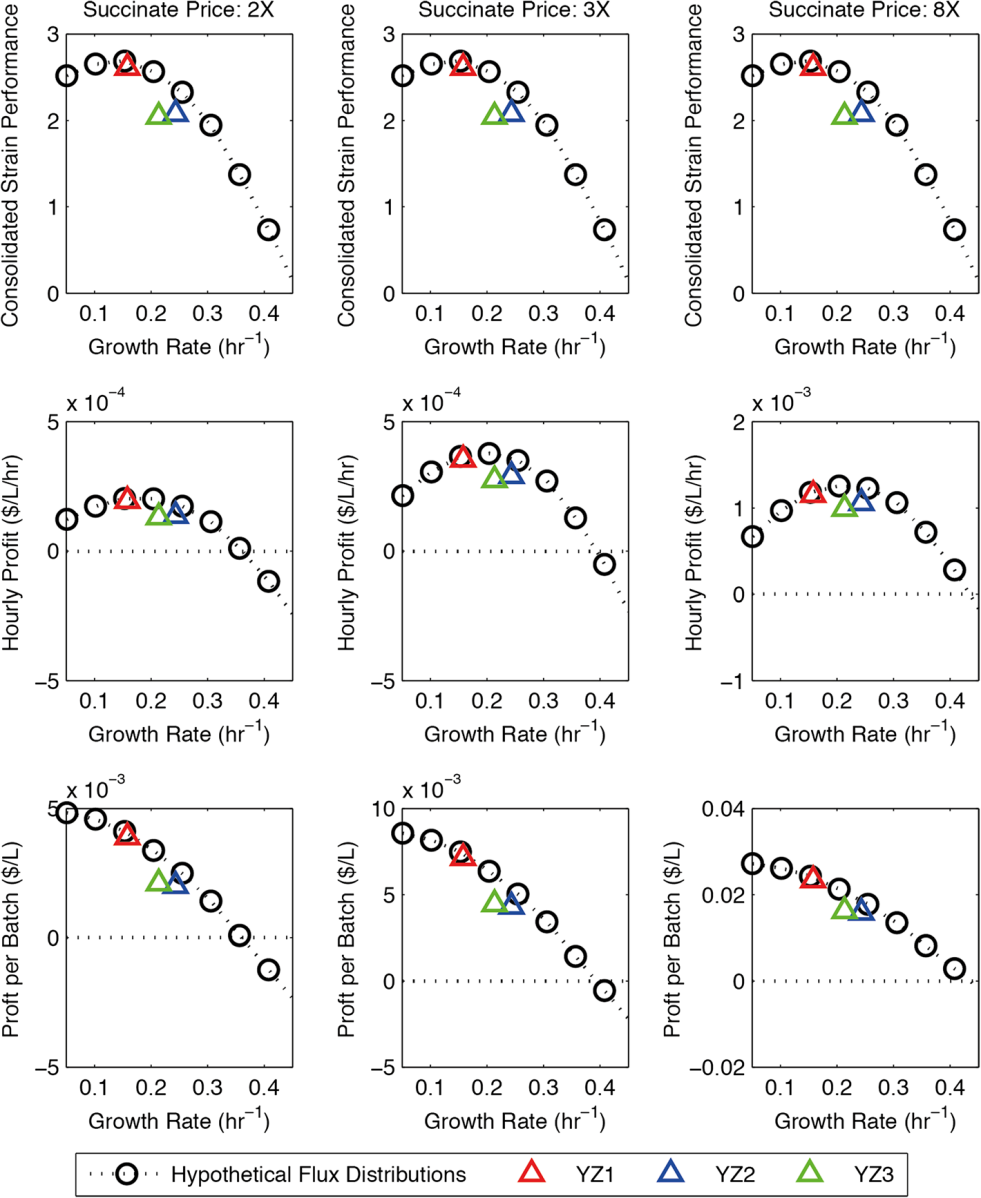
**The economic performance of the BDO-producing strains.** The consolidated strain performance **(A, B, C)**, hourly profit **(D, E, F)**, and profit per batch **(G, H, I)** of the BDO-producing strains at three different BDO price points. The horizontal dotted line **(D-I)** indicates the zero profit line.

Similar to the succinate case, during the “scanning” phase, ten hypothetical flux distributions were created along the BDO-production envelope. Interesting, the slope of BDO-production envelope (Figure 4B) is much less than that of the succinate envelope (Figure 2B), indicating that the tradeoff between the growth yield and the product yield is less severe in the BDO case. As a result, whereas a clear optimal growth rate range is observable in the succinate case (Figure 2B, Figure 3A-C), the optimal range is much less obvious in the BDO case (Figure 4B, Figure 5A-C). We rejected the hypothetical flux distributions growing at 0.05 hr^−1^ because it heavily favors product yield over productivity, and selected the large range of 0.1-0.35 hr^−1^ for strain design (Figure 3A).

During the “design” phase, two strains were selected from the list of strains produced by GDLS, hereby designated as “YZ4”, “YZ5”. YZ4 has a growth rate of 0.3 hr^−1^ and a product yield of 0.52 mol/mol. YZ5 has a growth rate of 0.35 hr^−1^ and a product yield of 0.51 mol/mol (Table 2). We have also recreated the knockout scheme used by Yim *et al.* (2011) in the iAF1260 model for comparison. Interestingly, this strain based in iAF1260 (designated as YIM1260) behaved differently than the strain reported by Yim *et al.,* which is based on an older model (designated as YIM^gm^). Whereas YIM^gm^ grew at lower rate (0.14 hr^−1^) and had a high yield (0.76 mol/mol), YIM^1260^ grew at higher rate (0.30 hr^−1^) and had a lower yield (0.52 mol/mol).

**Table 2:** Knockout strategies for BDO overproduction identified in this work

**BDO Strains**	**YZ4**	**YZ5**	**YIM**^**1260**^
Growth rate (hr^-1^)	0.3	0.35	0.3
Product yield	0.52	0.51	0.52
(mol/mol glc)			
Knockouts	ALCD2x	ALCD2x	ALCD2x
	PFL	PFL	PFL
	PGI		MDH
	TKT2		LDH_D

The knockouts are listed by their reaction names in the iAF1260 genome-scale model of *E. coli* metabolism.

The two BDO strains identified in this work (YZ4 and YZ5, Table 2) showed similarities in the predicted flux distributions as YIM1260. Namely, all three strains used pyruvate dehydrogenase and the oxidative TCA cycle, and secreted acetate, all at similar levels. However, unlike YIM^1260^, in which malate dehydrogenase (MDH) is deleted, YZ4 and YZ5 utilize MDH in the reverse direction. FBA simulations predict that the knockout of MDH in YZ5 reduces BDO yield by 97% while increasing the activity of lactate dehydrogenase (LDH) in the reverse direction. In order to synthesize BDO, LDH must also be deleted, which leads to the construction of strain YIM^1260^ (Table 2). Both MDH and LDH activities are coupled to NADH consumption, and their deletion results in a loss of NADH sinks. To restore redox balance, alternative NADH-consuming reactions must become active, and the BDO synthesis pathway includes four NADH-consuming reactions. While channeling excess NADH to the BDO synthesis pathway in this manner improves BDO yield, this is achieved at the cost of lowered growth rate. In contrast, strain YZ5 increases growth rate at the cost of lowered product yield, through reverse MDH activity.

During the “selection” phase, dFBA was used to predict the maximum-achievable yield, titer, and volumetric productivity of the strains YZ4, YZ5, and YIM^1260^. Their performance is compared with the hypothetical flux distributions (Figure 4A, 5A-C). YZ5 had better performance than both YZ4 and YIM^1260^, making it the best of the designed strains; however, it still performed slightly worse than the several hypothetical flux distributions (Figure 4A, 5A-C). Interestingly, YZ5 is created by knocking out pyruvate formate lyase (PFL), alcohol dehydrogenase (ALCD2x) pathways, which is a subset of the knockouts of YIM^1260^, which included the knockout of malate dehydogenase (MDH) and lactate dehydrogenase (LDH_D) in addition to pyruvate formate lyase and alcohol dehydrogenase. In other words, YZ5 achieved a slightly better consolidated performance than YIM^1260^ (Figure 4A, 5A-C) using two less knockouts (Table 2).

### Succinate production in fedbatch

During our design of both the succinate and the BDO producing strains, we evaluated the strains’ performances in batch reactors. The DySScO strategy can also be applied to design strains used in fedbatch reactors. However, to do so, it is important to optimize the feeding strategy as it can greatly influence the outcome. Ideally, a bilevel optimization problem can be formulated to simultaneously optimize the strain design and the feeding strategy; however, this is a difficult computational problem. Since our goal was to demonstrate the necessity and effectivness of a dynamic strain design strategy, we simplified the problem by adopting an idealized feeding strategy in which the glucose concentration is maintained at a high level (20mM) until the reactor becomes full. Using dFBA, we simulated the dynamics of all the hypothetical flux distributions and designed succinate-producing strains in this ideal fedbatch reactor.

We found that strains’ product yields are similar in both batch and fedbatch reactors (Figure 2B, 6B). While the titer and productivity are significantly higher in the fedbatch reactor, the trends between strains are similar (Figure 2C,D, 6C,D). As a result, the consolidated strain performance scores of the strains are similar between the two reactors. The 0.15 hr^-1^ strain remains the best hypothetical flux distributions, and YZ1 remains the best designed strain.

**Figure 6:**
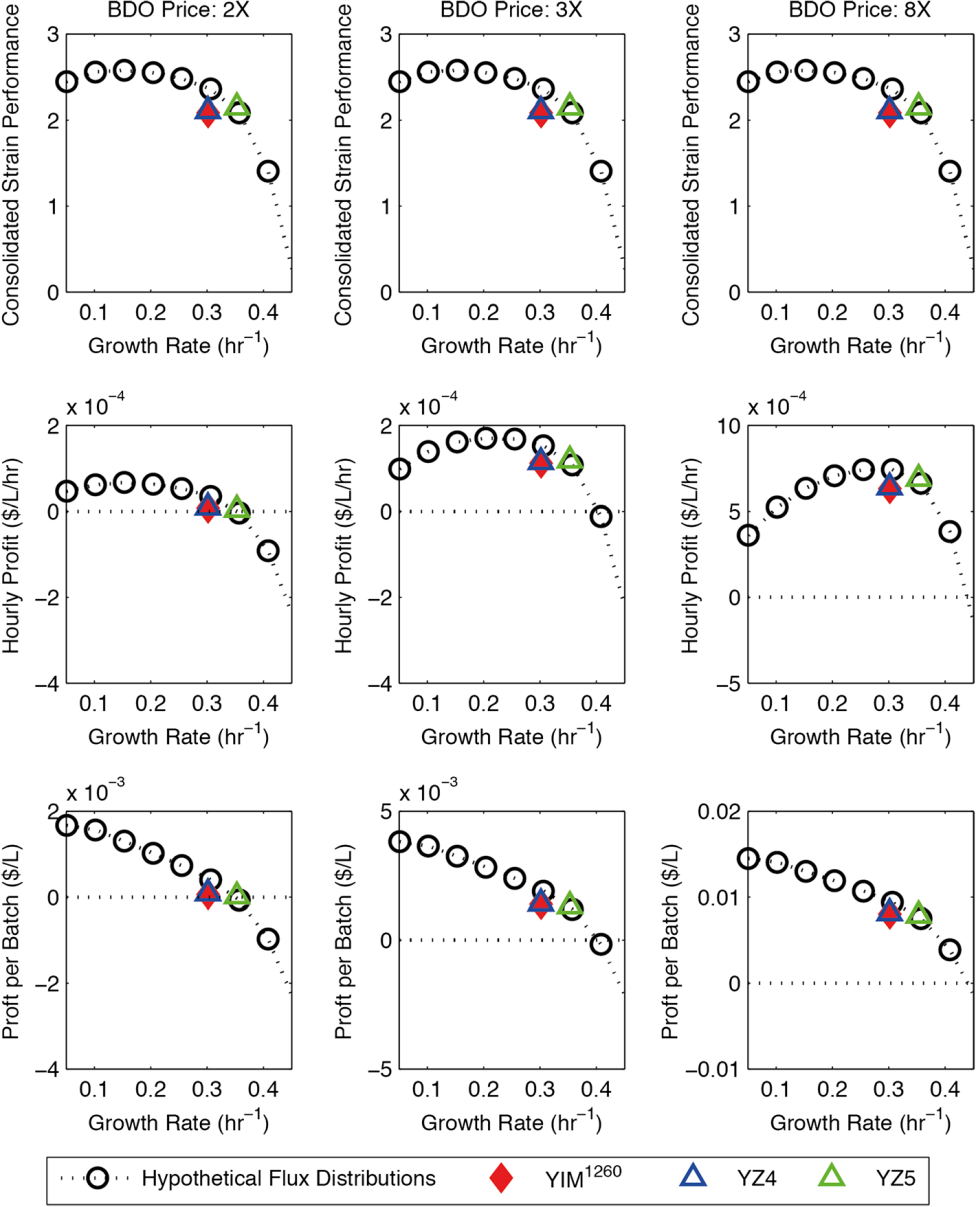
**The performance of the succinate-producing strains in fed-batch. (A-D)** The consolidated strain performance **(A)**, succinate yield **(B)**, volumetric productivity**(C)**, and titer **(D)** of both the hypothetical flux distributions and the designed strains in fed-batch simulations. The red dotted line indicates the range of growth rates selected for the static strain design process. The simulations assume the implementation of an ideal feeding strategy where the glucose concentration is maintained at 20 mM until the reactor is full. **(E)** The tradeoff between the succinate yield and the volumetric productivity. The Pareto frontier is outlined by the dotted line connecting the hypothetical flux distributions.

However, it is important to note that the titer of the strains is predicted to reach 1200 mM, which is unrealistic as the growth of *E. coli* becomes inhibited at high acid concentrations. It was recently reported that *E. coli* strains NZN111, AFP111, and BL21 can tolerate up to 680 mM of succinate [32], a concentration similar to the predicted titer of the fast-growing strains, but much higher than the predicted titer of the slow-growing strains (Figure 6D). As such, it seems that this inhibitory effect will significantly reduce the consolidated performance of the slow-growing, high yield strains in some situations, making such strains less valuable. However, it should be noted that succinate titer will not reach this level if a constant feeding strategy is adopted instead of an idealized exponential feeding strategy. Nevertheless, if the succinate inhibition kinetics are available, it can be incorporated into the DySScO formulation (See Method, Additional file 1: Figure S1).

### Balancing growth yield and product yield

The main motivation behind the development of the DySScO strategy is our recognition that an increased product yield does not necessitate an increase in titer or productivity, a recognition rooted in the known tradeoff between growth yield and product yield. Since a higher growth yield also lead to a higher growth rate at a given substrate uptake rate, the tradeoff between growth and product yields is reflected in the tradeoff between the growth rate and the product yield (Figure 2B, 4B). Our simulations confirmed that this basic tradeoff between growth rate and product yield gives rise to three additional tradeoffs: the tradeoff between growth rate and productivity (Figure 2C, 4C), the tradeoff between growth rate and titer (Figure 2D, 4D), and the tradeoff between the product yield and productivity (Figure 2E, 4E).

It is important to note that whereas the growth rate *vs.* product yield tradeoff and the growth rate *vs.* titer tradeoff have linear shapes (Figure 2B,D, 4B,D), the yield *vs.* productivity tradeoff and the rate *vs.* productivity tradeoff both have a parabolic shape (Figure 2C, 4C). This means that there is a growth rate at which the volumetric productivity can be maximized, at least theoretically. Since the consolidated strain performance is first-order linear combination of the normalized yield, titer, and productivity, it also has a hyperbolic shape and has a theoretical maximum.

Equally important to product yield and productivity is the titer. In industrial settings, the titer often governs the downstream process costs such as the cost of separating the desired chemical from the byproducts. In our simulations, the titer of a strain generally mirrors the product yield of the strain (Figure 2B,D, 4B,D), except at very low growth rates (Figure 4B,D). This is because we used a fast growing microorganism and allowed unlimited batch times, allowing all the substrate to be used for fermentation. However, if the strains were limited by their batch time, slower growing strains would be at a disadvantage with respect to titer. As an example, we compared the behaviors of two strains inside a fedbatch reactor, growing at 0.1 hr^−1^ and 0.25 hr^−1^ respectively. Given 5 days, the 0.1 hr^−1^ strain will produce higher titer than the 0.25 hr^−1^ strain; however, if the batch time is limited to 2 days, the 0.25 hr − 1 strain will produce higher titer than the 0.1 hr − 1 strain (Additional file 1: Figure S2). As such, there exist a potential tradeoff between growth rate and titer if batch time is a limiting factor. This tradeoff becomes significant from a batch scheduling perspective, and can be evaluated by observing the product titer dynamics predicted by DySScO (Additional file 1: Figure S2).

### Analyses of the economic viability of the strains

The production of chemicals through biological means from biomass offers many social and ecological benefits in comparison to conventional petrochemicals [35]. However, a basic requirement of a successful bioprocess is that it must be economically viable. Since the yield, titer, and productivity of the strains can be calculated from the simulated process dynamics, the economic viability of the overall bioprocess can be evaluated using various financial metrics. In this proof of principle study, we evaluated the economic performance of the strains in the batch reactor using two simple economic metrics – the hourly profit (profit made per hour, Equation 9) and the batch profit (profit made per batch, Equation 10). These metrics were calculated at three different price points – when the product price is at 2X, 3X, and 8X the price of the feedstock – and compared them to the consolidated strain performance scores (Figure 3, 5).

The hourly profit is a measure of the economic throughput of bioprocess. It is influenced by both the yield and the productivity of the strain, and is optimized at the medium growth rate range (Figure 3D-F, 5D-F). On the other hand, the batch profit is a measure of the profit generated per unit of feedstock. It is influenced by the titer and the yield, and is optimized at very low growth rates (Figure 3G-I, 5G-I). Whereas the consolidated strain performance (Figure 3A-C, 5A-C) is completely unaffected by the price point, the hourly profit and the batch profit are greatly affected by the price point. In addition, whereas the batch profit always favors high-yield (slow growing) strains, the hourly profit favors high yield strains at lower price points, and favors high productivity strains at higher price points. This effect is very prominent in the BDO-producing strains: whereas the hourly profit is optimized near 0.15 hr^-1^ at the 2X price point, it is optimized near 0.3 hr^-1^ at the 8X price point (Figure 5D-F), which is near the productivity optimal point (Figure 4C).

It is important to note that there is a tradeoff between the two economic metrics – a strain that maximizes the hourly profit will produce mediocre batch profit (Figure 3d-I, 5D-I). The relative importance of these metrics depends on higher-level design objectives: *ie.* whether to maximize economic throughput or the economic return on feedstock investment. Nevertheless, a strain is only economically viable if both the hourly profit and the batch profit are above zero. In a real world engineering setting, additional costs of the bioprocess, such as separation cost, should be included in the analysis. This zero-profit line is indicated by the horizontal dotted line (Figure 3d-I, 5D-I). As the price point drops, this line rises in relation to both the hourly profit and the batch profit curves, making higher growth rate strains economically unviable. At the price points tested, all the strains we designed (YZ1, YZ2, YZ3, YZ4, YZ5) were viable, although the BDO strains (YZ4, YZ5) produced very little profit at the 2× price point. In addition, the hourly profit of the BDO strains is very sensitive to the price point. For example, a process utilizing YZ5 is barely viable at the 2× price point becomes highly profitable at the 8× price point from an hourly profit perspective. This result demonstrates that in general, the economic performance of a strain may be highly sensitive to the price of the product.

One significant caveat of this economic viability analysis is that neither metrics took downstream processing costs and equipment depreciations into consideration. Nevertheless, despite the simplicity of the metrics, the results are both useful and illuminating. In particular, strain designs with poor economic performance can be eliminated early in the development cycle. Additionally, in applications where the engineering goal is to maximize profitability, financial metrics can replace the consolidated strain performance as the strain selection criteria in Steps 4 and 9 of the DySScO algorithm (see Method). The ability to perform such economic assessment is a distinct benefit of the DySScO strategy.

### Assumptions and limitations

A key assumption we made in this work is that the flux balance analysis (FBA) method is able to quantitatively predict the growth rate and the by-product secretion rates with sufficient accuracy. An underlining assumption of FBA is that the cellular objective is the maximization of biomass growth. It has been shown that alternative formulations, such as the minimization of metabolic adjustment [36], regulatory on-off minimization [37,38], and optimal resource allocation objective [39,40] can improve the predictive capability of the metabolic model under certain conditions. In particular, the minimization of metabolic adjustment and the regulatory on-off minimization methods may be more appropriate for simulating the metabolism of the knockout strains. To adopt these alternative formulations in DySScO, the equation 2d can be replaced by the appropriate formulation.

A major limitation of present work is that the substrate uptake rate must be fixed at a measured or assumed value in order to calculate the growth rate. Since the productivity and the titer are both functions of the growth rate, they are highly sensitivity to the changes in the substrate uptake rate. This is particularly problem organism such as *E. coli* has different substrate uptake rates in aerobic and anaerobic conditions. Recently, Zhuang *et al.* have demonstrated that the inclusion of a novel membrane occupancy constraint in FBA can be used to predict the growth rate without a fixed substrate uptake rate, and this method can be used to explain and predict the substrate uptake rate changes in aerobic and anaerobic conditions [29]. Further investigation is needed to determine if the inclusion of this constraint can help improve the DySScO method.

### Alternative approaches

During the development of the DySScO strategy, we have considered a slightly different strategy. First, we would generate a large number of strain designs using exiting algorithms; then, we would evaluate the dynamic properties of these strains using dFBA. Although this approach is slightly more straight-forward than the DySScO strategy, it is much more time consuming. By scanning the production envelope first for an optimal growth rate range, we were able to significantly reduce the time required to find the optimal strain. Our experience is that the advantage of the DySScO strategy is more obvious in cases where the tradeoff between growth yield and product yield is more severe.

Anesiadis *et al.* (2008) suggested that the tradeoff between growth yield and product yield can be balanced using a genetic toggle-switch: a strain can be developed to grow fast initially (making little or no product) and once sufficient biomass is produced, a genetic switch is triggered to cause the strain to stop growing and focus on chemical production [41]. This is an interesting alternative approach, and should be investigated alongside DySScO in the future.

## Conclusion

The first model-based strain-design algorithm, OptKnock, was developed a decade ago [8]. Since then, numerous strain-design algorithms with expanded capacity and improved computational time have been developed [9-11,14,42]. Unfortunately, despite the impressive innovations in these algorithms, they are limited by the stoichiometric nature of the metabolic models – without the addition of a process model, these algorithm are incapable of using dynamic design criteria such as titer and productivity. This is a significant limitation from an industrial perspective because the economic value of a strain is evaluated by the combination of yield, titer, and productivity. We addressed this limitation by integrating the dFBA method with the existing strain design algorithms to form the DySScO strategy.

In this proof of concept study, we applied the DySScO strategy to the problems of designing succinate-producing and BDO-producing *E. coli* strains. We evaluated the relationship between the growth yield, growth rate, product yield, volumetric productivity, and titer, as well as the economic viability of the designed strains. We showed that the economic performance of a strain can be strongly affected by the price difference between the product and the feedstock. As petroleum price continue to rise, commodity chemical prices is likely to increase as well, making more bioprocesses economically attractive. In the future, it would be interesting to develop a framework that integrates the DySScO strategy with financial projection models, thereby evaluating the economic potential of the strain. This kind of innovation will help broaden the application of model-based strain-design in the industrial context.

## Competing interests

The authors declare that they have no competing interest.

## Authors' contributions

KZ conceived the DySSco framework, performed simulations, analyzed data and wrote the paper, LY performed the strain designs, analyzed data and wrote the paper, WC analyzed data and wrote the paper, KM conceived the idea, analyzed data, and wrote the paper. All authors read and approved the final manuscript.

## Supplementary Material

Additional file 1: Figure S1 Succinate inhibition modeling. The liquid volume in the reactor (A), the biomass concentration (B), and the glucose (C) and succinate (D) concentrations of the succinate-producing strain YZ1 modeled with and without succinate-inhibition. Figure S2. Titer *vs.* Fed-batch Time. The predicted succinate production dynamics of the strains growing at 0.1 hr^-1^ and 0.25hr^-1^ are shown. The titer of the strain (illustrated by the circles) is defined as the concentration of succinate at the end of fed-batch time. If the fed-batch time is long (eg. 120 hrs), then the higher yield 0.1 hr^-1^ strain will have a higher titer. On the other hand, if the fed-batch time is short, the faster growing 0.25 hr^-1^ strain will have a higher titer. (DOCX 197 kb) Click here for file
