# Supplementary material for: Dynamic strain scanning optimization: an efficient strain design strategy for balanced yield, titer, and productivity. DySScO strategy for strain design
Source: BMC Biotechnol. 2013 Feb 6;13:8. doi: 10.1186/1472-6750-13-8 (PMC3574860; doi:10.1186/1472-6750-13-8)
Supplement: Additional file 1: Figure S1 — Succinate inhibition modeling. The liquid volume in the reactor (A), the biomass concentration (B), and the glucose (C) and succinate (D) concentrations of the succinate-producing strain YZ1 modeled with and without succinate-inhibition. Figure S2. Titer vs. Fed-batch Time. The predicted succinate production dynamics of the strains growing at 0.1 hr-1 and 0.25hr-1 are shown. The titer of the strain (illustrated by the circles) is defined as the concentration of succinate at the end of fed-batch time. If the fed-batch time is long (eg. 120 hrs), then the higher yield 0.1 hr-1 strain will have a higher titer. On the other hand, if the fed-batch time is short, the faster growing 0.25 hr-1 strain will have a higher titer. (DOCX 197 kb) [file 1472-6750-13-8-S1.docx]

SUPPLEMENTARY INFORMATION


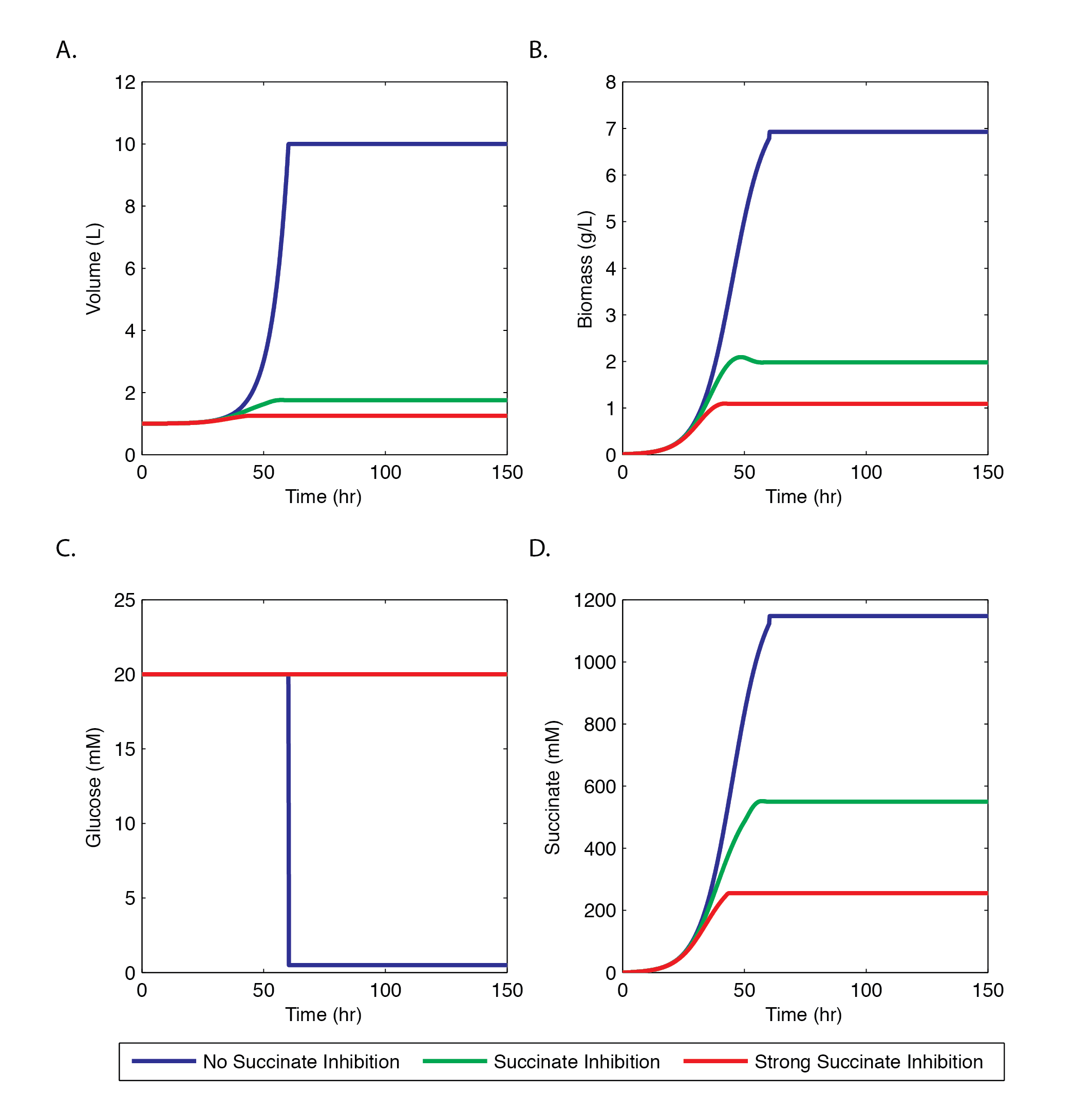


Figure S1. Succinate inhibition modeling

The liquid volume in the reactor (A), the biomass concentration (B), and the glucose (C) and succinate (D) concentrations of the succinate-producing strain YZ1 modeled with and without succinate-inhibition.

In our idealized fed-batch, without succinate inhibition modeling (blue lines), the model predicts the rapid production of biomass (B) as well as succinate (D) until the bioreactor becomes full (A), at which point glucose is no longer added to the system. If succinate inhibition is modeled (red and green lines), the cell growth is much slower (B) and less succinate is produced (D). The cell growth terminates at the critical succinate concentrations (B), the reactor is not filled and the glucose is never fully consumed (D). The final succinate titer is much lower if the succinate inhibition effect is included.


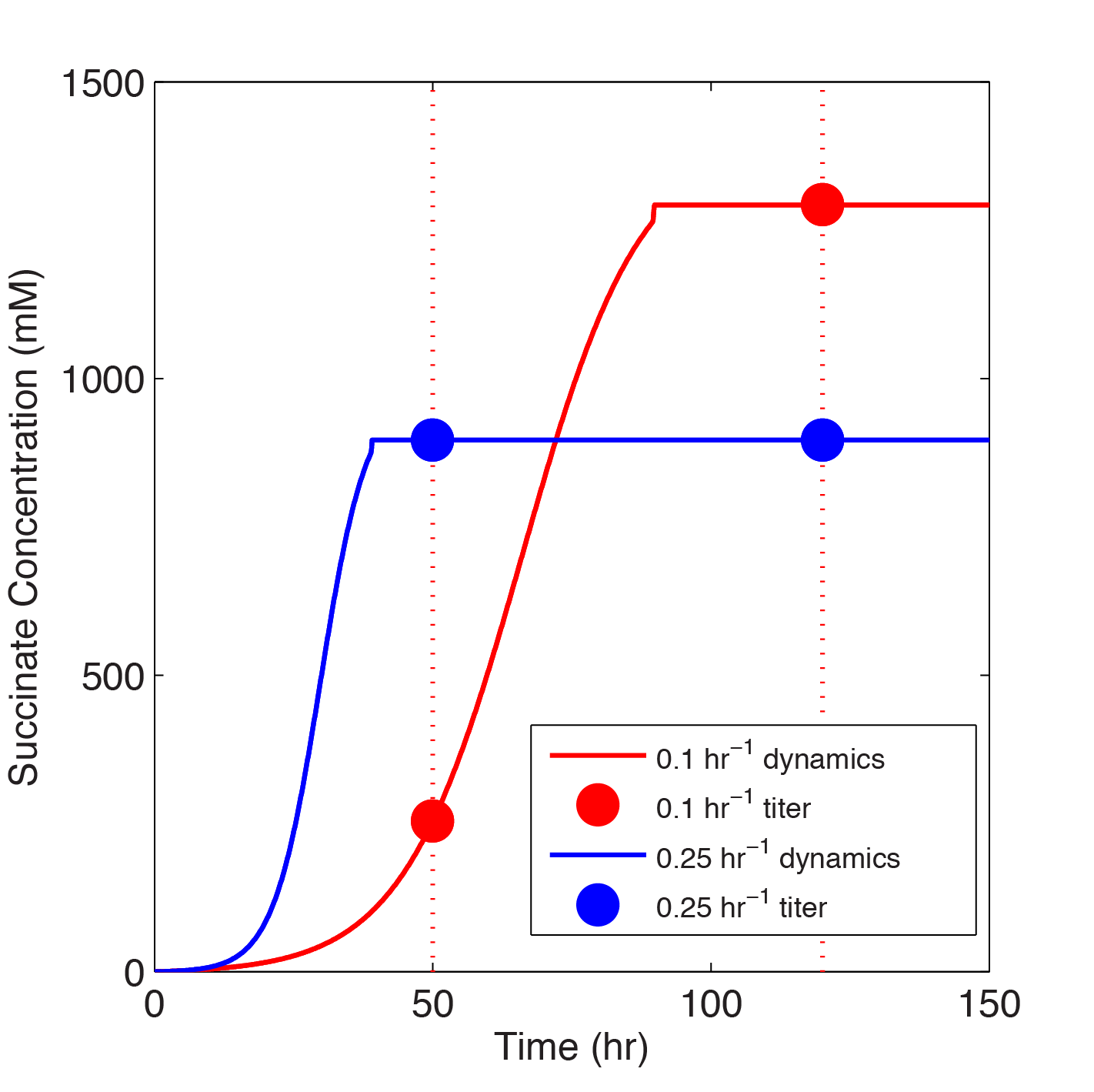


Figure S2. Titer vs. Fed-batch Time

The predicted succinate production dynamics of the strains growing at 0.1 hr^-1^ and 0.25hr^-1^ are shown. The titer of the strain (illustrated by the circles) is defined as the concentration of succinate at the end of fed-batch time. If the fed-batch time is long (eg. 120 hrs), then the higher yield 0.1 hr^-1^ strain will have a higher titer. On the other hand, if the fed-batch time is short, the faster growing 0.25 hr^-1^ strain will have a higher titer.
